# P-655. The Relative Risk of Acute Cardiopulmonary Events Following Hospitalization with Respiratory Syncytial Virus or Human Metapneumovirus Infection: A Modified Self-Controlled Case Series Analysis

**DOI:** 10.1093/ofid/ofaf695.868

**Published:** 2026-01-11

**Authors:** Lisa Glasser, Corey Fang, Chengbin Wang, Renee Gennarelli, Kainan Sun, Casey A Dobie, Xiaohui Zhao, Aimee Near

**Affiliations:** AstraZeneca, Wilmington, DE; AstraZeneca, Wilmington, Delaware; AstraZeneca, Wilmington, Delaware; Cencora Inc., Conshohocken, Pennsylvania; IQVIA, Plymouth Meeting, Pennsylvania; Cencora Inc., Conshohocken, Pennsylvania; IQVIA, Plymouth Meeting, Pennsylvania; IQVIA, Plymouth Meeting, Pennsylvania

## Abstract

**Background:**

Respiratory syncytial virus (RSV) and human metapneumovirus (hMPV) are common causes of respiratory infections. Sharing similar pathogenesis, both viruses cause significant morbidity and mortality in older adults. While RSV infection has been documented to increase the risk of acute cardiopulmonary events in adults, contemporary real-world data post-COVID-19 pandemic on RSV and similar data on hMPV are limited.

**Figure 1.**
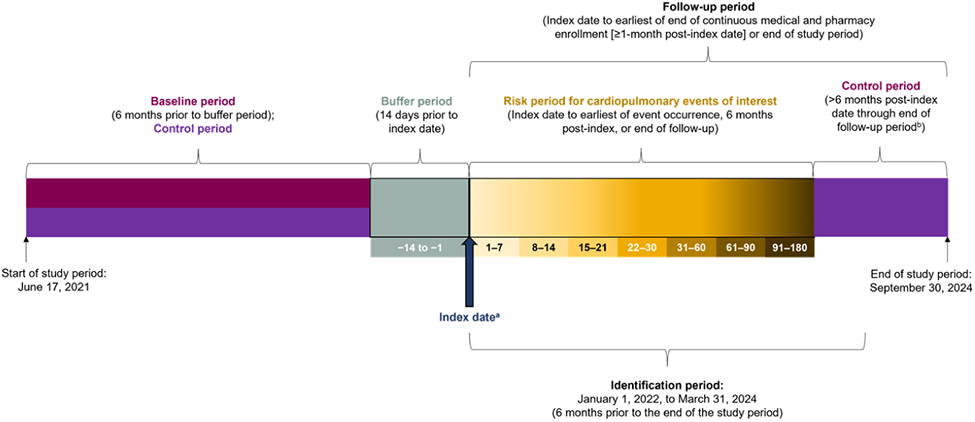
Study designa The study employed a modified self-control case series design, in which all patients who had the exposure (i.e., hospitalization with ICD-10-CM-coded RSV or hMPV) were analyzed, and the time periods of interest (control, buffer, risk) were anchored on the exposure. This modified approach minimizes selection bias and more accurately reflects the true underlying risk in the exposed population. It also enhances the generalizability to the exposed population by not limiting analysis to patients with cardiopulmonary events of interest. Those without any observed events were also included in the analysis. hMPV=human metapneumovirus; ICD-10-CM=International Classification of Diseases, 10th revision, Clinical Modification; RSV=respiratory syncytial virus. a. Earliest claim for hospitalization with ICD-10-CM-coded RSV or hMPV during the identification period. b. Patients were followed until the earliest of first hospitalization with an ICD-10-CM code of the index pathogen (RSV or hMPV), plan disenrollment, or the end of study period.

**Table 1.**
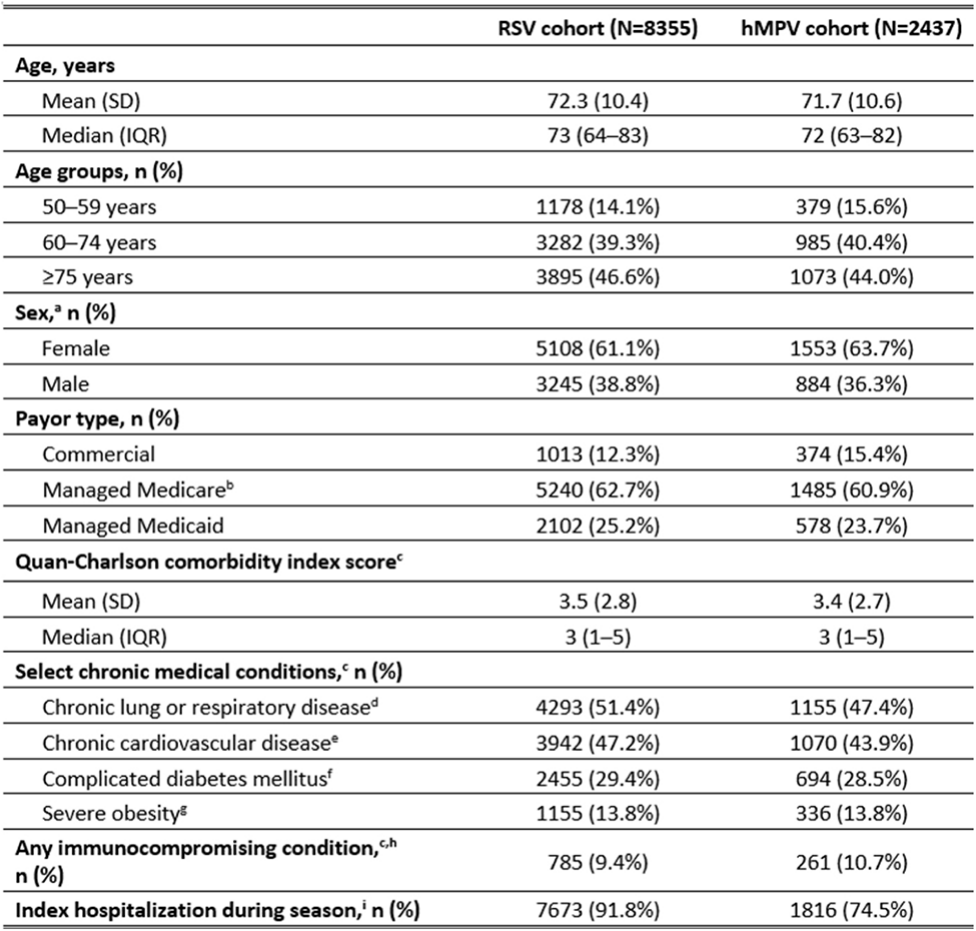
Demographic and baseline characteristics of the RSV and hMPV cohorts at index date CAR=chimeric antigen receptor; CKD=chronic kidney disease; COPD=chronic obstructive pulmonary disease; HIV=human immunodeficiency virus; hMPV=human metapneumovirus; ICD-10-CM=International Classification of Diseases, 10th revision, Clinical Modification; IQR=interquartile range; RSV=respiratory syncytial virus; SD=standard deviation. a. There were 2 patients in the RSV cohort with unknown/missing sex. b. Plans included Medicare Advantage and Medicare Supplemental; no Medicare Fee-for-Service plans were included. c. Identified in the 6-month baseline period (i.e., day −15 to day −194). d. Identified by ≥1 ICD-10-CM diagnosis code for chronic bronchitis, emphysema, COPD, asthma, bronchiectasis, interstitial lung diseases, chronic respiratory failure, or cystic fibrosis. e. Identified by ≥1 ICD-10-CM diagnosis code for chronic ischemic heart disease, heart failure, or congenital heart disease. f. Identified by ≥1 ICD-10-CM diagnosis code for diabetes complicated by CKD, neuropathy, retinopathy, or other end-organ damage. g. Identified by ≥1 ICD-10-CM diagnosis code for severe obesity (body mass index ≥40 kg/m2). h. Identified by ICD-10-CM, national drug codes, and/or Healthcare Common Procedure Coding System codes for hematologic malignancy, solid tumor malignancy with active treatment, hematopoietic stem cell transplant, receipt of CAR T-cell therapy infusion, receipt of islet cell or solid organ transplant with active anti-rejection therapy, moderate-to-severe primary immunodeficiency, advanced HIV disease, or active treatment with B-cell targeted therapies. i. For the study, RSV season was defined as October 1st through March 31st and hMPV season was defined as February 1st through July 31st.

**Methods:**

A modified self-controlled case series study was designed as in Fig 1. Adults aged ≥ 50 years hospitalized with RSV or hMPV between January 1, 2022, and March 31, 2024, were identified from IQVIA PharMetrics® Plus claims data and assigned to RSV or hMPV cohorts, with earliest admission date as the index date. The buffer period was 14 days pre-index date, the risk period included the first 180 days post-index date, and the control period included the 180 days before the buffer period and all time after the risk period. Patients included had complete follow-up pre-index date and at least 1-month post-index date. Incidence rate ratios (IRRs) for acute cardiopulmonary events comparing the incidence rates of buffer and risk periods to that of the control period were calculated using multivariable conditional Poisson regression models.

**Figure 2.**
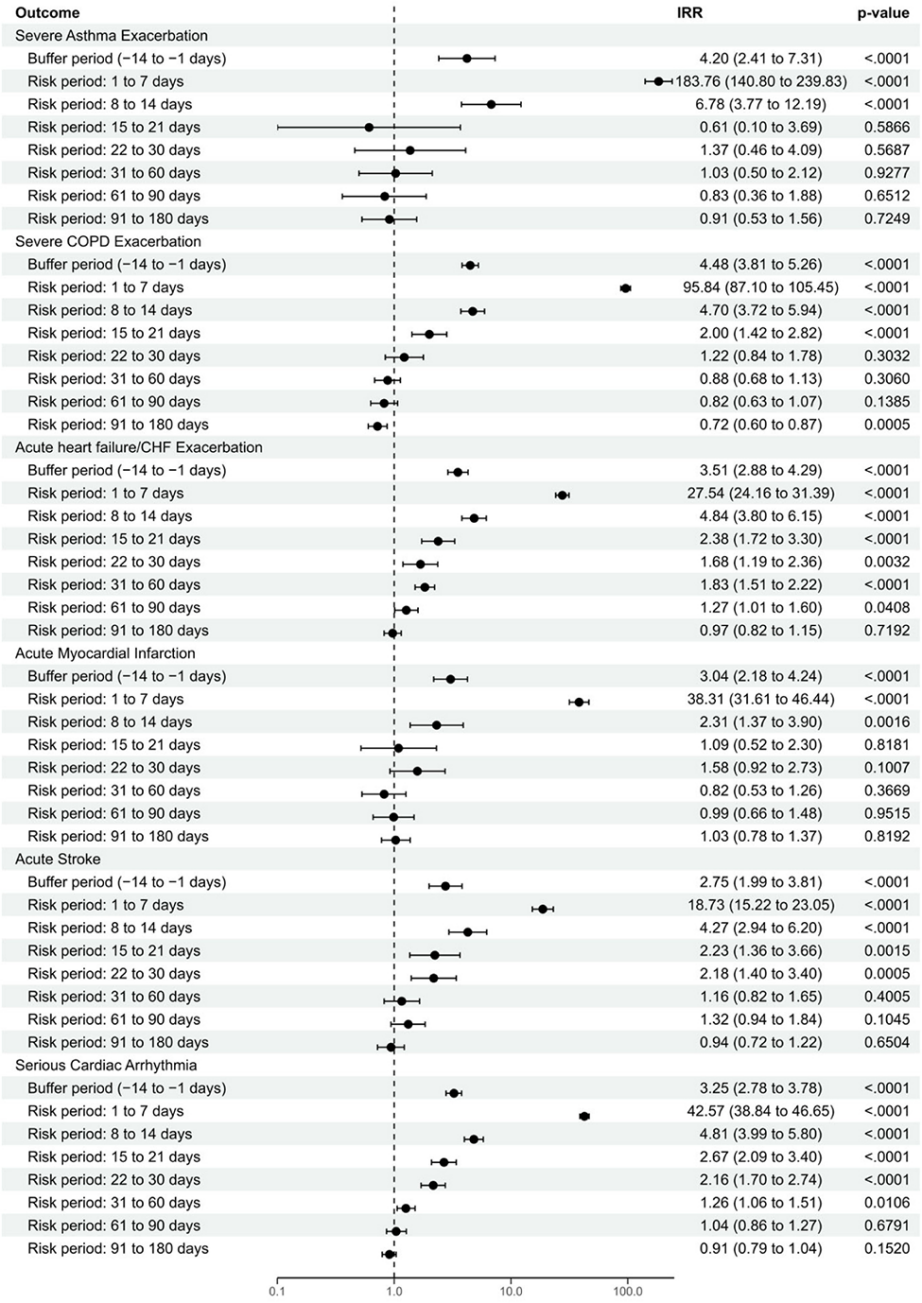
Incidence rate ratios for acute cardiopulmonary events during or following admission with ICD-10-CM-coded RSV p<0.05 denotes statistical significance. IRRs were not estimable in cases where no patients experienced the event during the risk period. CHF=congestive heart failure; COPD=chronic obstructive pulmonary disease; ICD-10-CM=International Classification of Diseases, 10th revision, Clinical Modification; IRR=incidence rate ratio; RSV=respiratory syncytial virus.

**Figure 3.**
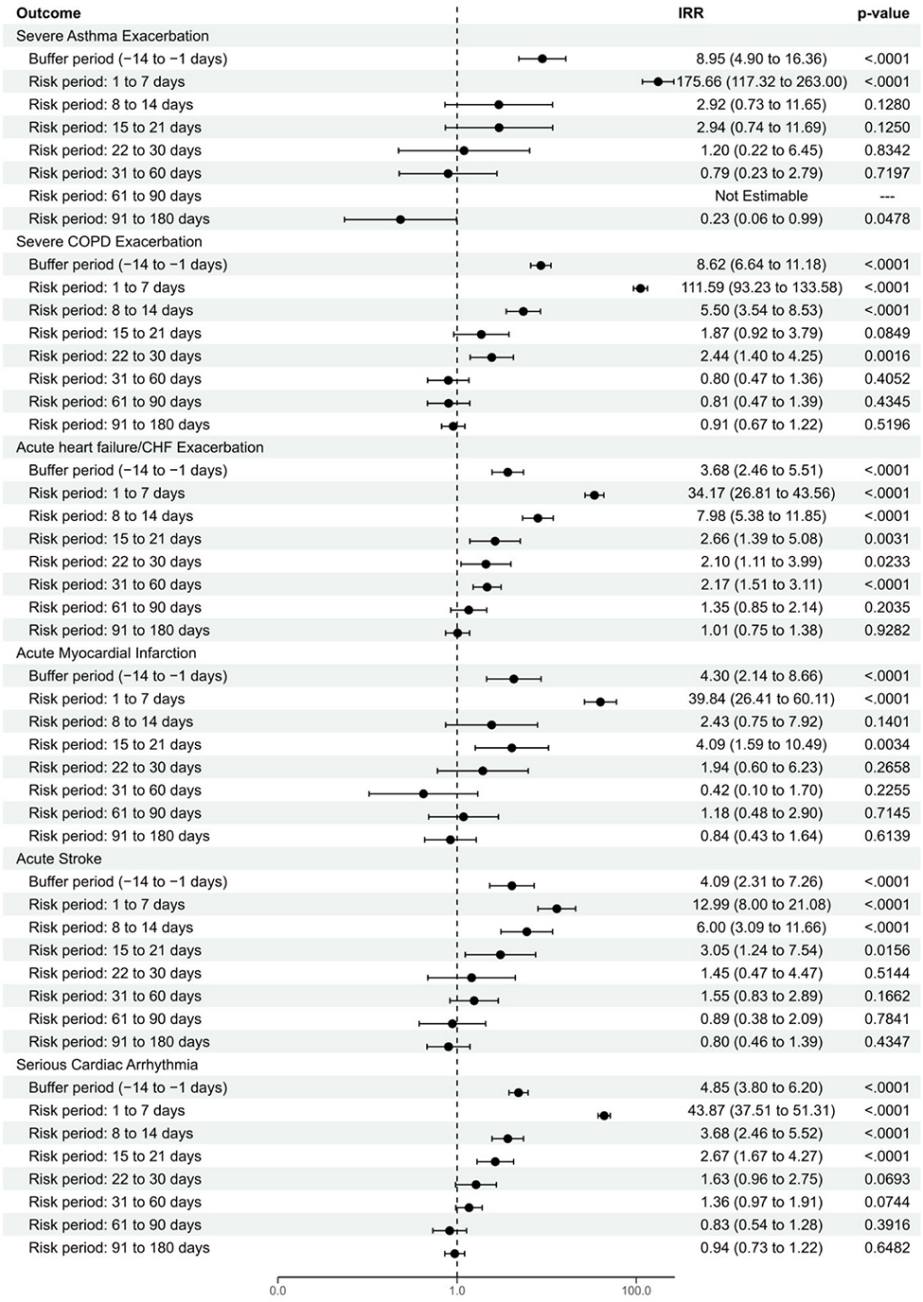
Incidence rate ratios for acute cardiopulmonary events during or following admission with ICD-10-CM-coded hMPV p<0.05 denotes statistical significance. IRRs were not estimable in cases where no patients experienced the event during the risk period. CHF=congestive heart failure; COPD=chronic obstructive pulmonary disease; hMPV=human metapneumovirus; ICD-10-CM=International Classification of Diseases, 10th revision, Clinical Modification; IRR=incidence rate ratio.

**Results:**

Baseline characteristics are shown in Table 1. Significantly increased IRRs were observed for all cardiopulmonary events post-RSV or hMPV admission, peaking in the first week post-index (Fig 2–3). IRRs were significantly higher for 21 days post-index for most events in both cohorts, with the longest effect observed for acute heart failure/congestive heart failure exacerbation (90 days). A sensitivity analysis censoring at first event yielded similar results, confirming the robustness of our findings and event independence.

**Conclusion:**

Older adults hospitalized with RSV or hMPV infections have a significantly increased short-term risk of acute cardiopulmonary events, particularly in the first week following admission; the increased risk may remain up to 90 days. These results underscore the substantial clinical burden of severe RSV and hMPV infections and their associated complications, highlighting a critical need for preventive measures and targeted therapies to reduce hospitalizations in this vulnerable population.

**Disclosures:**

Lisa Glasser, MD, AstraZeneca: Stocks/Bonds (Public Company) Corey Fang, PharmD, AstraZeneca: Stocks/Bonds (Public Company) Chengbin Wang, MD, PhD, AstraZeneca: Stocks/Bonds (Public Company) Renee Gennarelli, MS, AstraZeneca: Advisor/Consultant Kainan Sun, MS, PhD, AstraZeneca: Advisor/Consultant Casey A. Dobie, PharmD, AstraZeneca: Advisor/Consultant Xiaohui Zhao, PhD, AstraZeneca: Advisor/Consultant Aimee Near, MPH, AstraZeneca: Advisor/Consultant

